# The Revised ACE Pyramid: A Contemporary Framework for Understanding Childhood Adversity and Advancing Toxic Stress Prevention and Healing

**DOI:** 10.3390/children13091261

**Published:** 2026-09-17

**Authors:** Krista Kotz, Rachel Gilgoff, Andy Krackov, Bart Klika, Melissa Merrick

**Affiliations:** 1 University of California, Los Angeles-University of California, San Francisco (UCLA-UCSF) ACEs Aware Family Resilience Network (UCAAN), University of California, Los Angeles, CA 90024, USA; rachelgilgoffmd@gmail.com (R.G.); andy@hillcrestadvisory.com (A.K.); 2 The D*Stress Foundation, San Francisco, CA 94105, USA; 3 Sean N. Parker Center for Allergy and Asthma Research, Stanford University, Palo Alto, CA 94304, USA; 4 Prevent Child Abuse America, Chicago, IL 60602, USA; bklika@preventchildabuse.org (B.K.); mmerrick@preventchildabuse.org (M.M.)

**Keywords:** Adverse Childhood Experiences (ACE), childhood adversity, toxic stress, prevention, trauma-informed, public health, health equity, Positive Childhood Experiences (PCEs), protective factors, safe, stable, nurturing relationships and environments (SSNREs)

## Abstract

**Highlights:**

**What are the main findings?**

- The revised ACE Pyramid incorporates more than two decades of advances in the science of toxic stress while preserving the familiar structure of the original framework.
- The revised framework positions prolonged or excessive activation of the stress response as a central mechanistic pathway linking childhood adversity to lifelong health outcomes.

**What are the implications of the main findings?**

- The framework supports a shift from viewing ACEs primarily as risk indicators toward understanding and addressing toxic stress biology as a target for prevention and intervention.
- It provides a shared framework for informing pediatric practice, public health, research, and policy aimed at improving outcomes for children and families.

**Abstract:**

**Background:** The Adverse Childhood Experiences (ACE) Pyramid has been widely used for more than two decades to illustrate pathways linking childhood adversity to health outcomes across the lifespan. Since its development, substantial advances in the science of toxic stress have expanded understanding of the biological mechanisms through which adversity influences lifelong health. **Objective:** To describe the development and scientific rationale for a revised ACE Pyramid that reflects contemporary evidence on toxic stress, the biological pathways linking childhood adversity to health, and opportunities for prevention and healing. **Methods:** A multidisciplinary revision process was conducted in collaboration with subject matter experts from government, academia, healthcare, professional associations, and community-based organizations. Iterative review and refinement were used to align the framework with current scientific evidence and practice. **Results:** A revised ACE Pyramid was developed that preserves the familiar structure of the existing framework while incorporating advances in the science of toxic stress, prevention, and healing. Compared with the ACE Pyramid featured on the CDC website during development of the revised framework, the revised framework more explicitly emphasizes prolonged or excessive activation of the stress response as a central mechanistic pathway linking childhood adversity to lifelong health, expands the biological layer to encompass multiple interacting biological systems beyond neurodevelopment alone, and integrates prevention and healing opportunities throughout the framework. **Conclusions:** The revised ACE Pyramid synthesizes more than two decades of advances in the science of toxic stress into an accessible conceptual framework. It explains how childhood adversity becomes biologically embedded to shape lifelong health while emphasizing that prevention is possible and healing can occur throughout the life course. By providing a shared framework for understanding toxic stress biology, it may help inform trauma-informed clinical care, public health action, research, and policy aimed at advancing health equity and improve outcomes for children and families.

## 1. Introduction

In 1998, results from a seminal study on Adverse Childhood Experiences (ACEs) were published by researchers from Kaiser Permanente (KP) and the Centers for Disease Control and Prevention (CDC). This retrospective study of approximately 17,300 adults demonstrated that ten categories of childhood abuse, neglect, and household challenges were common, with 64% of participants reporting exposure to at least one ACE during the first of 18 years of life. The study also identified a graded relationship between the number of ACE categories experienced and multiple health risk factors and diseases [1,2,3].

Since publication of this initial study, a robust body of research demonstrates that ACEs are associated with many common and serious health and life conditions [1,3,4,5,6,7]. ACEs are linked to at least nine of the ten leading causes of death in the United States, including heart disease, cancer, unintentional injury, stroke, chronic lower respiratory diseases, Alzheimer’s disease, diabetes, kidney disease, and chronic liver disease and cirrhosis, as well as high-school non-completion, adult unemployment, and poverty status as an adult [2,5,6,7,8,9].

**Figure 1:**
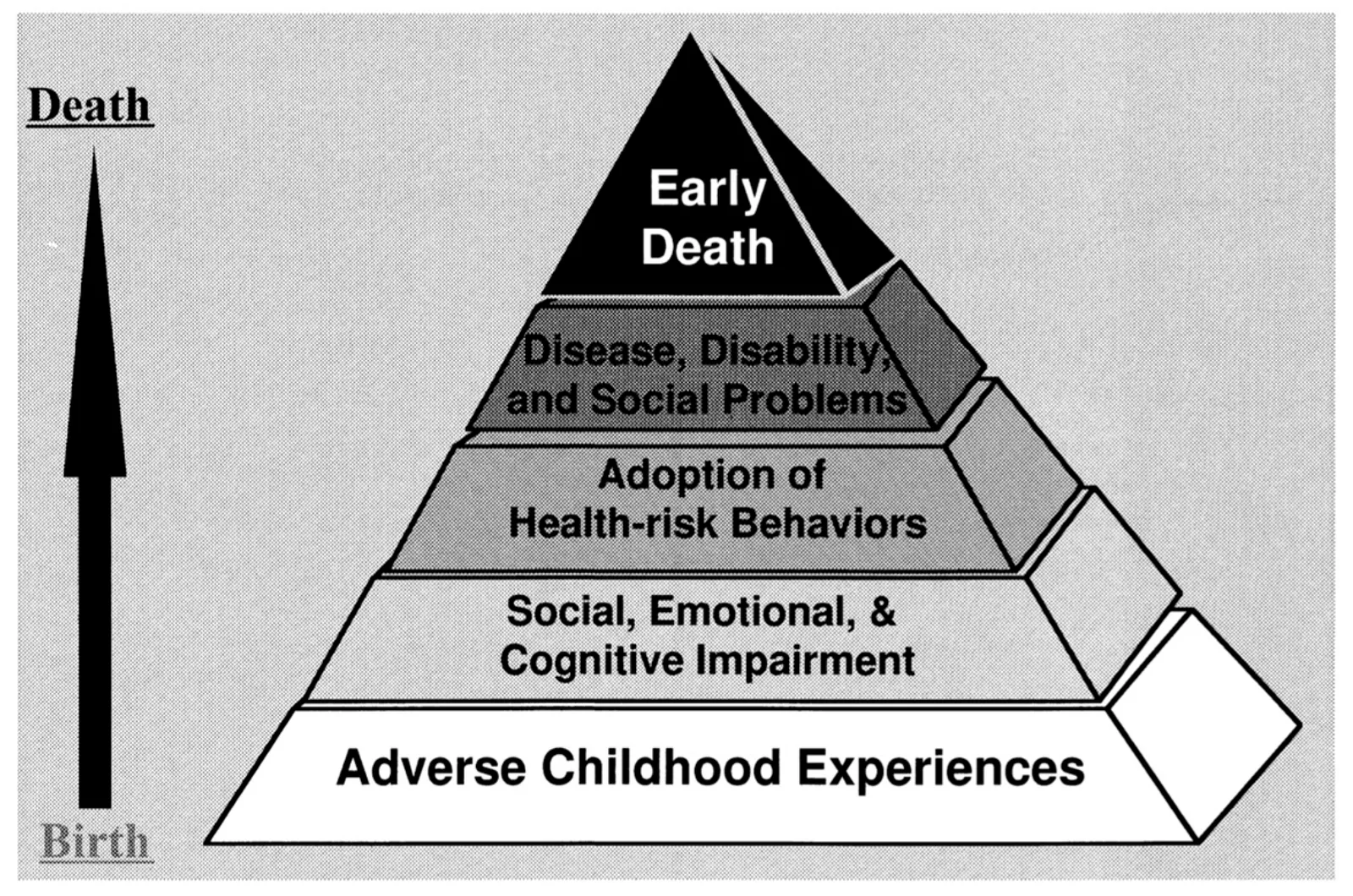
Original ACE Pyramid published in the landmark 1998 study. Reprinted from Felitti et. Al. “Relationship of Childhood Abuse and Household Dysfunction to Many of the Leading Causes of Death in Adults.” *American Journal of Preventive Medicine*, 14, 245–258, with permission from Elsevier.

**Figure 2:**
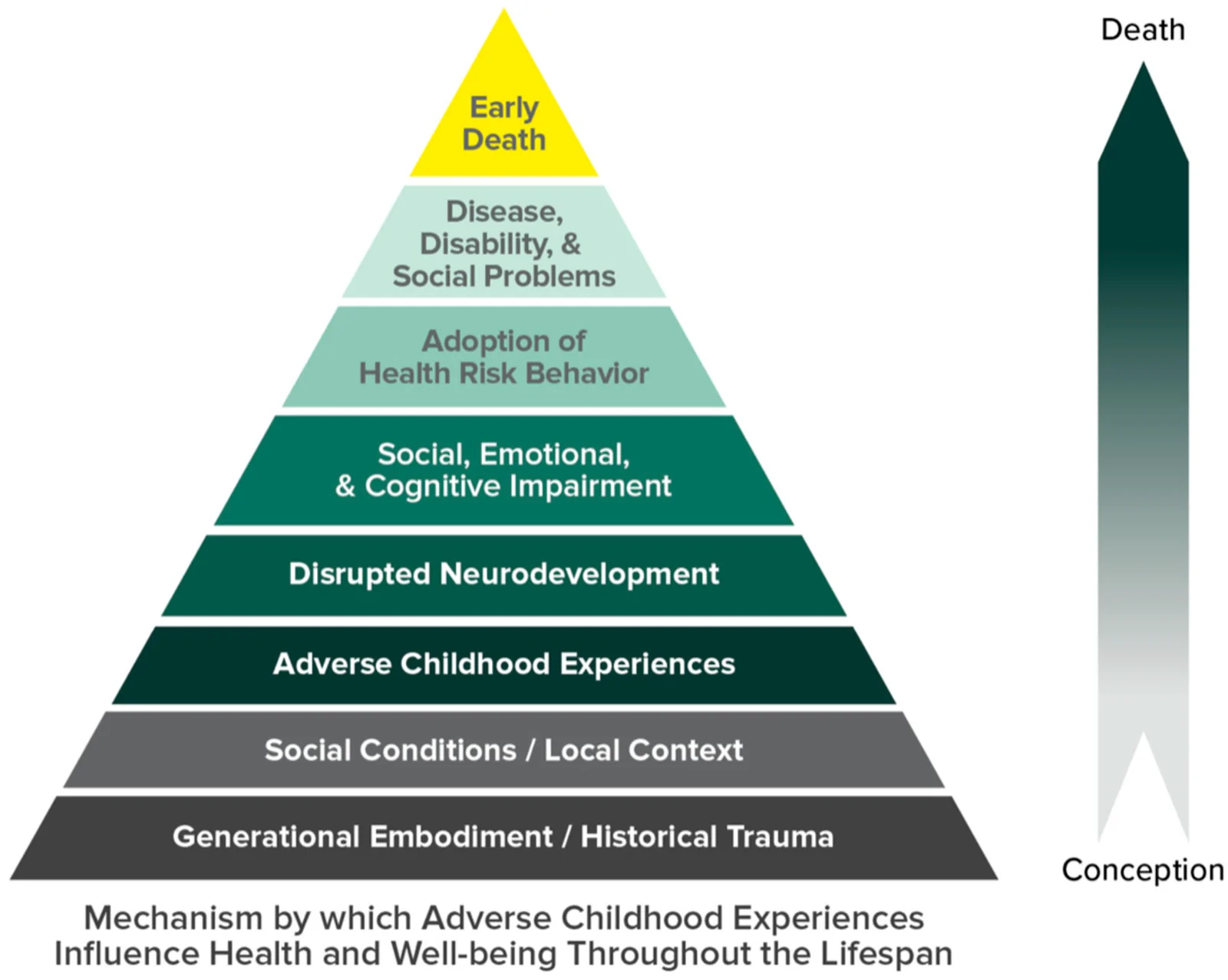
ACE Pyramid featured on the CDC ACEs website during development of the revised framework.

When the original KP–CDC study was first published, the authors proposed that ACEs contribute to poor health outcomes primarily through their influence on health risk behaviors, such as smoking or substance misuse, which may function as neuroregulatory coping mechanisms [2]. An image of this conceptual pathway was first depicted in the original 1998 study (see Figure 1) [2]. As understanding of the impacts of early life adversity on the developing brain expanded [10], along with recognition of the systemic and structural factors that contribute to disproportionate exposure to risk and opportunity [11], a later version added two rows below ACEs, Generational Embodiment/Historical Trauma and Social Conditions/Local Context, and one row above ACEs, Disrupted Neurodevelopment. This version was featured on the CDC’s ACEs website during the development of the revised framework (see Figure 2) [12].

While the original ACE Pyramid remains an important conceptual framework for understanding the relationship between childhood adversity and health, subsequent research has substantially expanded the understanding of the mechanisms linking early adversity to health. Growing evidence highlights the physiological effects of chronic, excessive, or severe stress responses on lifelong physical, mental, and social health—especially when such stress occurs during sensitive periods of child development [4,13,14,15]. The revised ACE Pyramid retains this overarching framework while incorporating an updated model of the biological pathways through which adversity becomes biologically embedded and influences lifelong health. This new graphic also expands the framework by explicitly integrating opportunities for prevention and healing.

## 2. Materials and Methods

An ACE Pyramid revision team was convened, in partnership with CDC colleagues leading national ACEs-related efforts, to explore updating the existing graphic and to develop a process for getting input from leaders in the field. The revision process aimed at aligning the new pyramid with contemporary evidence on the impacts of early life adversity. Although alternative visual frameworks from the scientific literature were considered, each presented limitations related to complexity, accessibility, or ease of adoption. Given the widespread familiarity and use of the existing ACE Pyramid, the decision was made to retain the pyramid format to support continuity, usability, and broad dissemination.

**Figure 3:**
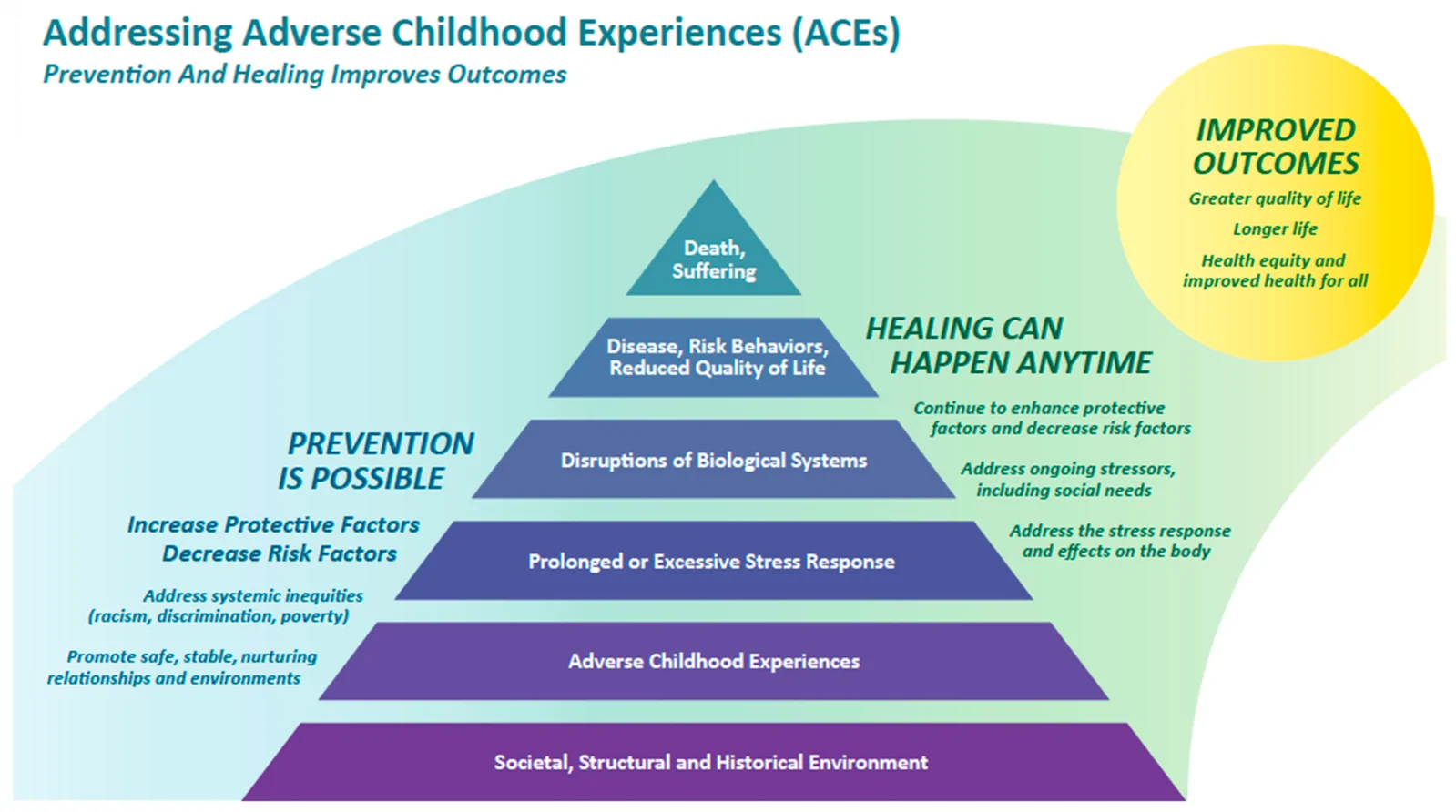
Revised ACE Pyramid.

The revision process began by articulating a clear project goal: to develop an updated ACE Pyramid graphic that illustrates the mechanisms by which ACEs influence health across the lifespan (see Figure 3). To support this goal, the team developed a process grounded in the latest scientific evidence and informed by broad multidisciplinary partner input. The revision process prioritized areas in which there was substantial and converging evidence supporting updates to the conceptual framework. Partners and subject matter experts with backgrounds relevant to ACEs, childhood adversity, toxic stress, prevention, healthcare, public health, and related fields were invited to review and provide feedback on successive versions of the framework. Contributors represented government, academia, healthcare, professional associations, and community-based and nonprofit organizations at the local, state, and national levels. Feedback was primarily obtained through virtual meetings, allowing contributors to discuss the conceptual content and visual representation of the framework with the revision team. Additional feedback was provided through written comments and other communications. Feedback was gathered over multiple stages of development, with revised versions of the pyramid subsequently refined and redistributed for additional input. At some stages, contributors were invited to provide broad feedback on the framework; at others, discussion focused on specific components or areas under revision. Some contributors provided feedback at multiple stages of the process, while others contributed at a single point in time.

Input and recommendations were tracked across review cycles, using spreadsheets, meeting notes, and other written records. Feedback was reviewed throughout the revision process and considered in relation to the scientific evidence, the goals and scope of the framework, and input received from other contributors. When differing perspectives arose, they were explored through discussion with contributors when feasible. Final decisions regarding whether and how feedback was incorporated were made by the two authors who led development of the revised framework (K.K. and R.G.). The process was iterative and consultative rather than a formal consensus process; formal consensus methods such as a Delphi procedure, voting, or predetermined thresholds for agreement were not used.

In total, 76 partners (including authors) from 44 organizations and affiliated institutions contributed content expertise or feedback at least once during the process and are listed in the Acknowledgments.

## 3. Results

The iterative revision process informed refinements to the content, terminology, organization, and visual representation of the framework, resulting in a revised ACE Pyramid (Figure 3) that preserves the familiar structure of the ACE Pyramid while incorporating contemporary scientific understanding of toxic stress, the biological pathways linking childhood adversity to health, and opportunities for prevention and healing.

Compared with the ACE Pyramid featured on the CDC website during the revision process (Figure 2), the revised framework more explicitly emphasizes prolonged or excessive activation of the stress response as a central mechanistic pathway linking childhood adversity to lifelong health. It also expands the biological layer to encompass multiple interacting biological systems beyond neurodevelopment alone and integrates prevention and healing opportunities throughout the framework.

The resulting framework comprises the following interconnected components: (1) societal, structural, and historical environment; (2) Adverse Childhood Experiences; (3) prolonged or excessive stress response; (4) disruptions of biological systems; (5) disease, risk behaviors, and reduced quality of life; and (6) death and suffering. Prevention and healing are represented across the framework rather than as a discrete sequential step, reflecting opportunities to alter pathways and improve outcomes across the life course.

## 4. Discussion

The revised ACE Pyramid provides a contemporary conceptual framework for understanding how childhood adversity influences lifelong health while highlighting opportunities for prevention and healing. As a conceptual framework, the pyramid illustrates key pathways and relationships but is not intended to imply that these pathways are uniformly linear or sequential. By integrating advances in the science of toxic stress with emerging knowledge of the biological pathways through which adversity becomes biologically embedded, the framework provides an updated conceptual foundation for understanding, preventing, and treating toxic stress across the life course.

Social, structural, and historical conditions shape children’s exposure to both risk and protective factors and can contribute to disproportionate exposure to Adverse Childhood Experiences (ACEs). Adverse Childhood Experiences are associated with a wide range of poor mental, social, and physical health outcomes [1,2,5,6,7]. The revised ACE Pyramid highlights a key mechanism linking ACEs to lifelong health: when childhood adversity leads to a prolonged or excessive activation of the stress response, the development of biological systems can be disrupted, affecting health across the life course [16].

### 4.1. Scientific Rationale for the Revised ACE Pyramid

The sections that follow discuss each component of the revised ACE Pyramid and summarize the scientific rationale supporting its inclusion.

#### 4.1.1. Societal, Structural and Historical Environment

Childhood development is shaped by societal, structural, and historical environments [4,17,18,19,20]. Children are exposed to a unique mix of factors that can either support or undermine health and well-being. Protective factors may include policies, systems, community conditions, and relationships that promote safety, stability, connection, and opportunity. Risk factors can include structural and historical injustices such as racism, discrimination, poverty, violence, and historical trauma, as well as policies or institutional practices that limit access to resources and opportunities necessary for healthy development.

Safe, stable, nurturing relationships and environments, along with other Positive Childhood Experiences are foundational for healthy child development and can help buffer the stress response [19,21]. At the same time, societal, structural, and historical stressors can increase the cumulative burden of adversity experienced by children and families and may reduce caregivers’ capacity to consistently provide these buffering relationships and environments. In this way, broader environmental conditions can increase risk for Adverse Childhood Experiences.

#### 4.1.2. Adverse Childhood Experiences

The term “ACEs” specifically refers to the 10 categories of adversities in three domains experienced by age 18 that were evaluated in the landmark KP-CDC study [1,2,3]:

- **Abuse:** Physical, emotional, or sexual;
- **Neglect:** Physical or emotional;
- **Household Challenges:** Incarceration, mental illness, substance misuse or dependence, parental separation or divorce, or intimate partner violence.

Children may also be exposed to other early life adversities not captured in the original ACEs study, such as poverty, racism, community violence, displacement, or other forms of social and structural adversity, which can also have negative long-term effects [4,17,18].

#### 4.1.3. Prolonged Excessive Stress Response

In response to ACEs and other early life adversity, some children will experience prolonged or excessive activation of their stress response system. Individual responses to adversity vary and may be influenced by genetic factors, developmental timing, prior experiences, and other biological and environmental factors. This might include the release of stress hormones (e.g., adrenaline, cortisol) and altered immune reactivity [13,16,17].

While the stress response is vital to health and survival, it is also a spectrum [14]:

- **Positive stress**: Mild or moderate and short-lived stress response necessary for healthy development.
- **Tolerable stress**: More severe stress response, but limited in duration or intensity, which allows for recovery.
- **Toxic Stress**: Extreme, frequent, or extended activation of the body’s stress response, resulting in changes in neurologic, endocrine, and immune system development.

#### 4.1.4. Disruptions of Biological Systems

Prolonged or excessive activation of the stress response, particularly during critical and sensitive periods, can disrupt the development of biological systems, including [13,14,16]:

- **Neurologic system:** A growing body of research is uncovering mechanistic pathways by which early life adversity can impact dysregulation across a number of neurologic systems: sensorimotor, arousal and energy, reward processing, autonomic nervous system, hypothalamic–pituitary–adrenal axis, cognitive processes, and attachment and relational neural networks [15]. Neurologic effects include brain volume changes in some regions, alterations in neural connectivity patterns, and disruptions of neurotransmitter function. This can increase risk for physical and mental health conditions such as chronic pain, sleep issues, addictions, blood pressure issues, anxiety, depression, and impairments in executive functioning, learning, and memory [15,22,23,24,25,26,27,28].
- **Endocrine system:** ACEs are associated with changes in the function of hormones, such as cortisol, growth hormone, thyroid hormone, and hormones associated with puberty increasing risk for growth issues, pubertal issues, weight changes, insulin resistance, and diabetes [13,15,29,30,31,32].
- **Immune function:** The acute stress response involves activation of the immune system to prepare the body in anticipation of potential injury. Prolonged activation of the stress response, however, can lead to dysregulation of the immune system, increased inflammatory markers with Th2-biased response, and increased risk for chronic inflammation, reduced immune function, or auto-immune disease [13,15,31,33,34].
- **Genetic regulatory:** Telomere erosion and epigenetic changes can increase risk for cancer, early mortality, and intergenerational transmission [13,15,31,35,36,37].
- **Attachment and relational**: Lack of safety and co-regulation from safe, stable, nurturing caregiver and environment can erode trust, self-esteem, and future relationships. Research has demonstrated disruptions in neural networks for mentalization, empathy, and protective hypervigilance which can lead to insecure attachment and social thinning [15,38,39,40].

#### 4.1.5. Disease, Risk Behaviors and Reduced Quality of Life

- **Disease:** Without intervention, the disruptions in biological systems (i.e., neurologic, endocrine, immune, genetic regulatory, and attachment and relational) can result in a wide range of physical and mental health problems in childhood and throughout adult life (for more detail on mechanistic pathways and disease implications ).

ACEs are associated with increased risk of over forty health conditions [4,5,6,7,13,41]. Some common and serious ACE-associated health conditions include cardiovascular disease, cancer, diabetes, and depression [1,2,5,6,7].

- **Risk Behaviors:** Disruptions in biological systems due to toxic stress can lead some people to adopt health risk behaviors, such as smoking, alcohol and other drug use, or overeating. This can happen in a couple of ways [4,23]:
  - Health risk behaviors are often adopted as coping strategies to feel better in the short term.
  - Toxic stress also alters reward pathways in the brain, which can result in adoption of health risk behaviors. The adoption of health risk behaviors is one pathway leading to disease. However, many of those who experience ACEs or other early life adversities do not adopt health risk behaviors yet still develop disease [42,43].
- **Quality of Life:** Disruptions in biological systems, such as executive functioning, mental health, or attachment systems (emotional bonding), can also lead to reduced quality of life—for example, reduced employment opportunities or income potential [9], as well as social thinning, loneliness, divorce or separation [44,45].

In addition to the pathway leading from excessive stress to biological disruptions to reduced quality of life, ACEs can influence health and life course outcomes through other psychosocial and contextual pathways. For example, ACEs can directly interfere with opportunities for educational experiences, healthy nutrition, and learning healthy relationship styles, which also can impact an individual’s quality of life [9,46,47].

#### 4.1.6. Death and Suffering

Without buffering support or intervention, disease, adoption of health risk behaviors and reduced quality of life can lead to suffering and early death [48,49]. In fact, people with six or more ACEs have been shown to die nearly 20 years earlier than those without ACEs (61 years vs. 79 years) [37].

#### 4.1.7. Prevention and Healing Improve Outcomes

Importantly, preventing and healing from ACEs are possible and can happen at any age. ACE exposure alone does not determine or foretell an individual’s future health or life outcomes. The balance of risk factors (e.g., societal, structural, and historical inequities and individual adversities) and protective factors (e.g., safe, stable, nurturing relationships, environments, and healing interventions) can influence whether an individuals will have positive or negative health outcomes. Together, through multidisciplinary and collaborative approaches, we can prevent ACEs, treat toxic stress, and improve health equity and health outcomes for all.

#### 4.1.8. Prevention Is Possible

Preventing early life adversity and toxic stress requires a comprehensive public health approach that strengthens the conditions in which families live, work, and raise children—particularly by addressing poverty, racism, and discrimination [50]. By increasing protective factors and decreasing risk factors, we can tip the scales toward prevention of ACEs and toxic stress [4].

Policy and systems changes, community-level efforts, and concrete strategies help dismantle structural barriers that disproportionately impact marginalized families and further build protective factors such as [4,51,52,53]:

- Expanding economic supports like paid family leave, flexible workplace policies, and tax credits like the Earned Income Tax Credit to reduce financial stress;
- Investing in evidence-based home-visiting programs like Healthy Families America, Nurse–Family Partnership and Parents as Teachers;
- Increasing access to parenting supports such as Triple P, Child–Parent Psychotherapy (CPP), Parent–Child Interaction Therapy (PCIT), and SafeCare;
- Equitable access to culturally responsive services such as mental health and substance abuse treatment and healthcare;
- Strengthening childcare standards and ensuring family access to high quality childcare centers;
- Providing mentoring and after-school programs;
- Career training and educational opportunities to support economic security;
- Disseminating public education campaigns that shift social norms from blaming parents for ACEs to recognizing ACEs as structural and systems issues [54].

Prevention is possible when we invest in strategies that alter the conditions and environments in which children and families live [50].

#### 4.1.9. Healing Can Happen Anytime

By addressing the external stressors and the internal stress biology we can help people heal from early life adversity.

Addressing stressors involves continued support in enhancing individual, systemic and structural protective factors and decreasing risk factors. This includes addressing individual basic needs such as housing and food insecurity and providing resources and crisis support if there are current, on-going ACEs or other adversities throughout the life course.

Addressing the internal stress response involves the use of healing strategies and interventions to address the neurologic, endocrine, immune, metabolic, and genetic regulatory effects that prolonged or excessive activation of the stress response can have on the body [21]. This includes evidence-based strategies such as safe, stable, nurturing relationships, physical activity, nutritional approaches and supplements, quality sleep, mindfulness practices, experiencing nature, and trauma-specific mental health interventions which have all been shown to regulate neuroendocrine, immune, and metabolic function [15,21,55]. For example, mindfulness practices have been shown to lower stress-related biomarkers including cortisol, C-Reactive Protein (CRP), blood pressure, and heart rate [55,56,57,58]. Spending time in nature has also been shown to decrease cortisol, blood pressure, and heart rate as well as improve cognitive functioning [59,60]. Parenting education programs program have been shown to improve parent–child relationships and child behavior and development, and a few have demonstrated reductions in child maltreatment [61]. In addition, trauma-responsive mental health interventions including trauma-focused cognitive behavioral therapy (TF-CBT), Eye Movement Desensitization and Reprocessing (EMDR), and Child–Parent Psychotherapy (CPP) can address nervous system dysregulation, negative cognitions, and attachment issues [62,63,64,65,66].

Through multidisciplinary and collaborative approaches, and ecosystems of support, we can prevent ACEs and heal from toxic stress, thus improving quality of life, length of life, health equity, and health outcomes for all.

### 4.2. Limitations

The revised ACE Pyramid is a conceptual framework grounded in contemporary scientific evidence and multidisciplinary partner and expert review; it has not yet undergone empirical evaluation as an integrated framework. Accordingly, the present work does not establish that the revised framework predicts health outcomes more accurately or improves clinical decision-making compared with previous versions of the ACE Pyramid or other conceptual models. Future research should evaluate its utility in clinical, public health, and research settings, including whether use of the framework improves understanding, assessment, intervention selection, or health outcomes.

The multidisciplinary revision process was iterative and consultative rather than a formal consensus-development process; formal consensus methods, such as a Delphi procedure or quantitative thresholds for agreement, were not used.

The pyramid format necessarily simplifies the complex pathways linking childhood adversity to health and life outcomes. These pathways are not uniformly linear or unidirectional, nor do all effects of childhood adversity occur through prolonged or excessive activation of the stress response. Individuals may experience different trajectories depending on the nature and timing of adversity and the interaction of biological, relational, social, and environmental risk and protective factors. The revised ACE Pyramid is therefore not intended to capture all possible pathways or interactions through which childhood adversity may influence subsequent layers of the framework, but rather to provide an accessible conceptual representation of key pathways supported by the current evidence.

The revised ACE Pyramid also does not address questions regarding the optimal conceptualization or measurement of childhood adversity, including differences in the type, frequency, intensity, duration, or developmental timing of adversity. The framework should not be interpreted as implying that different adversities have equivalent effects.

Finally, the revised ACE Pyramid was developed primarily within a U.S. context, and its applicability across different cultural and societal contexts has not been evaluated. The types of experiences considered adverse, as well as their meaning, impact, and the protective resources available to children and families, may vary across cultures and settings. Future research should examine the relevance and applicability of the framework across diverse cultural and international contexts.

## 5. Conclusions

The revised ACE Pyramid translates decades of advancing science into a practical, accessible framework intended to inform clinical care and public health action. By explicitly illustrating the biological pathways linking early adversity to lifelong health, the revised ACE Pyramid supports a shift from viewing ACEs solely as risk indicators to understanding and addressing toxic stress physiology as a treatable condition. In pediatric clinical practice, this framework may help inform trauma-informed care, screening and assessment, anticipatory guidance, and the integration of evidence-based interventions that regulate stress biology and strengthen protective factors. At the systems level, it can support alignment across healthcare, public health, and community sectors to build coordinated, prevention-oriented ecosystems that address both external stressors and internal stress responses.

Importantly, the revised ACE Pyramid highlights that these pathways unfold across the life course and across generations, underscoring the need for intergenerational approaches to care and prevention. Supporting the health and well-being of caregivers is foundational to improving outcomes for children, while early investment in children can alter trajectories for future generations. By integrating clinical, community, and policy strategies that strengthen safe, stable, nurturing relationships and environments, this updated framework can help advance health equity and improve outcomes for children and families. Overall, the revised ACE Pyramid provides a shared conceptual foundation for preventing and treating toxic stress in pediatric practice and beyond, reinforcing that prevention and healing are possible at any stage of life.

## Data Availability

The original contributions presented in this study are included in the article. Further inquiries can be directed to the corresponding author.

## Abbreviations

The following abbreviations are used in this manuscript:

ACE	Adverse Childhood Experiences
CDC	Centers for Disease Control and Prevention
CPP	Child–Parent Psychotherapy
CRP	C-Reactive Protein
EMDR	Eye Movement Desensitization and Reprocessing
KP	Kaiser Permanente
PCIT	Parent–Child Interaction Therapy
TF-CBT	Trauma Focused Cognitive Behavioral Therapy
